# Single Amino Acid-Based Control of Fibril Supramolecular Chirality

**DOI:** 10.1021/acs.jpclett.6c01104

**Published:** 2026-06-02

**Authors:** Jadon Sitton, Dmitry Kurouski

**Affiliations:** † Department of Biochemistry and Biophysics, 14736 Texas A&M University, College Station, Texas 77843, United States; ‡ The Institute for Quantum Science and Engineering, Texas A&M University, College Station, Texas 77843, United States

## Abstract

Supramolecular chirality determines the physiological function of biological macromolecules, including DNA and amyloid fibrils. However, factors that determine the supramolecular organization of such macromolecules remain poorly understood. In the current study, we demonstrate that the supramolecular chirality of amyloid fibrils can be controlled by a single amino acid and its protonation state. These findings are critically important for understanding the mechanism of self-assembly of functional and pathological amyloid aggregates.

## Graphical abstract

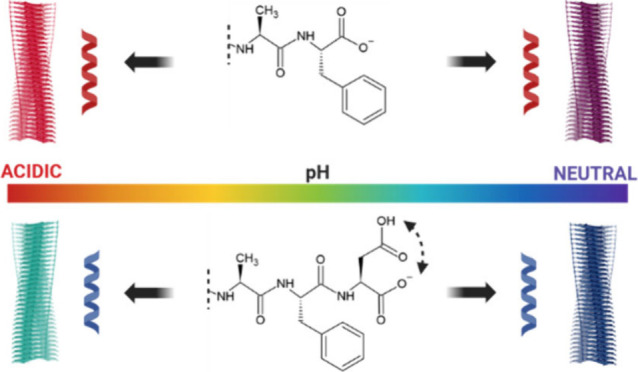

Absolute chiral configuration in low molecular weight compounds and supramolecular chirality of macromolecules have paramount importance for their biological properties. For instance, l-enantiomer of carvone, a natural monoterpene ketone found in essential oils of spearmint, gives minty taste, while its d-enantiomer has a spicy, rye-bread-like aroma and taste. Left-twisted Z-DNA, unlike its right-handed A and B forms, has a “zigzag” pattern which allows absorbing of the torsional strain produced by active genes. These supramolecular differences between different forms of DNA determine their biological (transcription) properties.

Pathological self-assembly of proteins results in the formation of long aggregates collectively known as amyloid fibrils.^1,2^ When proteins aggregate, they form β-sheets with 4.7 Å interstrand distances spaced ∼10 Å between each other.^3–5^ These β-sheets propagate in the direction perpendicular to plane of the strands forming filaments that braid and intertwine with other filaments forming fibrils.^6,7^ The free energy minimization makes fibrils twist adopting either right- or left-handed twists that can be visualized using electron (EM) or atomic force microscopy (AFM).^8–10^ In some cases, the twist becomes so tight that both EM and AFM are not capable of resolving it.^11,12^ This limitation can be overcome using vibrational circular dichroism (VCD).^12^ In 2007, Ma and co-workers showed that VCD could be used to probe supramolecular chirality of insulin and lysozyme fibrils based on the pattern of the amide I band, which primarily originates from carbonyl vibration of the peptide bond.^13^ Recently, our group found lipids and fatty acids, if present at the stage of protein aggregation, could reverse supramolecular chirality of insulin and lysozyme fibrils.^14,15^ Similar observations were previously made by Kurouski and co-workers for a large group of amyloidogenic proteins.^16^ The researchers observed that pH could be used to alter the supramolecular organization of amyloid fibrils. However, the exact molecular nature of this phenomenon remained poorly understood.

In this study, we employed two peptides with sequences RSFFSFLGEAF and RSFFSFLGEAFD, which have only one amino acid difference at the C terminus. These peptides originate from the N-terminus of serum amyloid A, a protein linked to the onset of secondary systemic amyloidosis.^17^ We first aggregated both RSFFSFLGEAF and RSFFSFLGEAFD at pH below pKas (2.1–2.2) of their C termini to remove possible contribution of charges present on the peptides (pH 1.5). Next, the peptides were aggregated at pH above p*K*_a_s (2.1–2.2) of their C termini (pH 3.0) and above the p*K*_a_ of aspartic acid (p*K*_a_ = 3.8) present at the C terminus of RSFFSFLGEAFD (pH 6.0). Finally, VCD and AFM were used to examine the morphology and supramolecular chirality of the peptide fibrils formed under all described experimental conditions. In VCD spectra, the amide I band is the marker of protein self-assembly.^13,16,18^ Its magnitude provides information about the magnitude of the protein self-assembly,^13^ while the sign of the amide I provides the information about the twist of the formed protein aggregates.^19,20^

We found that at pH 1.5, RSFFSFLGEAF formed fibrils that exhibit characteristic positive (1624 cm^–1^) and negative (1636 cm^–1^) bands in the amide I region (Figure 1). This vibrational pattern was previously reported for the left-twisted fibrils.^16,20,21^ Although AFM imaging did not reveal any twists on the fibril surface (Figure 2), previously reported EM analysis of RSFFSFLGEAF fibrils grown at pH 2.0 by Rubin and co-workers revealed the presence of left-twisted aggregates.^10^ We did not observe substantial changes in the pattern of the VCD spectra acquired from RSFFSFLGEAF fibrils grown at pH 3.0 (Figure 1). These results indicate that C-terminal deprotonation does not change the handedness of the peptide self-assembly. However, we observed a shift of a positive peak in the spectra acquired from the fibrils formed at pH 3.0 from 1624 cm^–1^ (pH 1.5) to 1616 cm^–1^ (Figure 1). These results suggest that deprotonation of the peptide C-terminus changes the fold of peptide backing in the fibrils. This fold change can be attributed to the additional electrostatic repulsion forces created by the negative charges present in the peptides. These conclusions are further supported by the observed difference in the height of these fibrils compared with the height of RSFFSFLGEAF fibrils grown at pH 1.5 (Figure 2). It should be noted that despite fold differences, RSFFSFLGEAF fibrils grown at pH 1.5 and pH 3.0 have the same secondary structure.This conclusion can be made from the same position of the amide I band (1624 cm^–1^) in the corresponding IR spectra (Figure 1).

**Figure 1:**
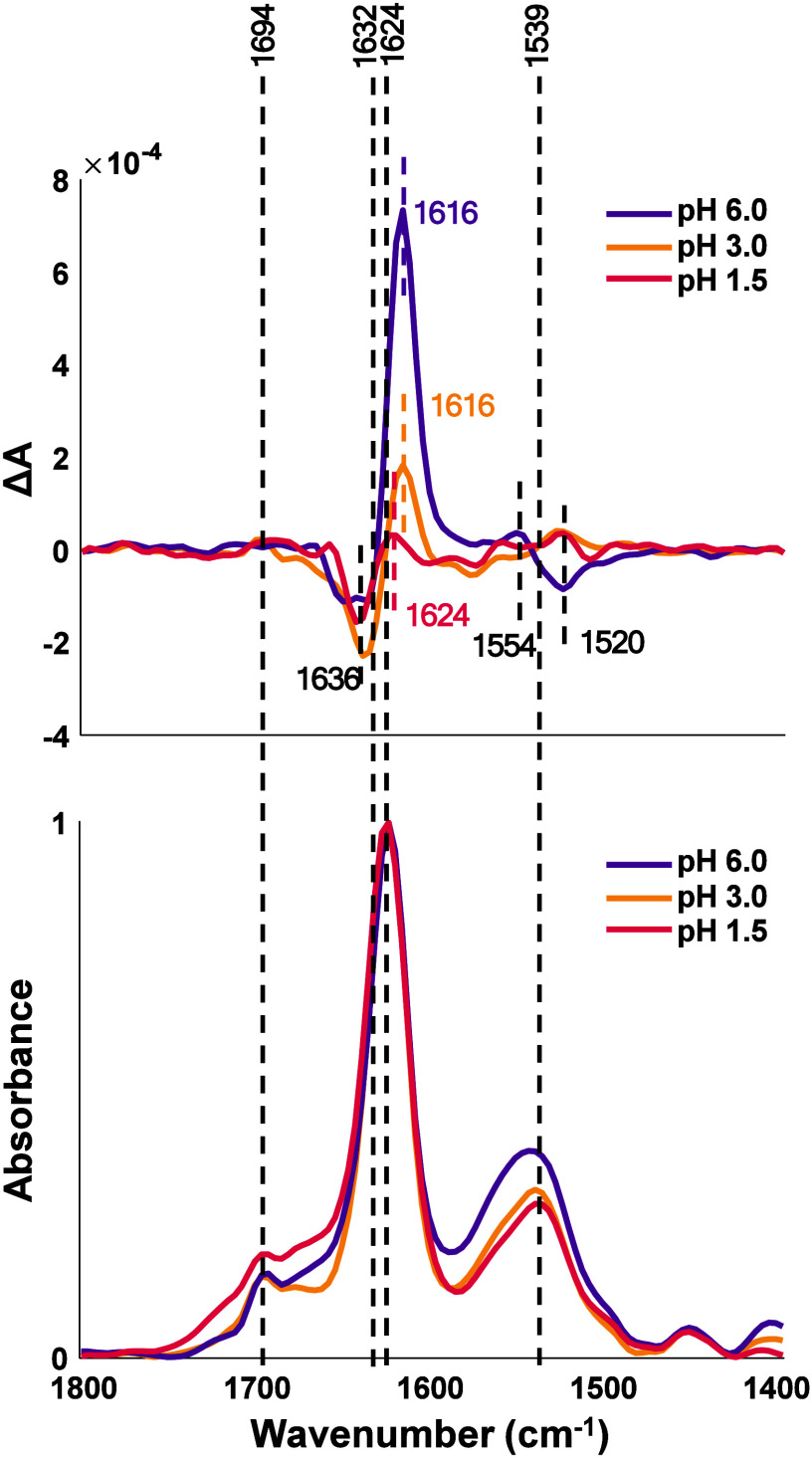
VCD (top) and IR (bottom) spectra acquired from RSFFSFLGEAF fibrils formed at pH 1.5 (red), 3.0 (yellow), and 6.0 (purple).

**Figure 2:**
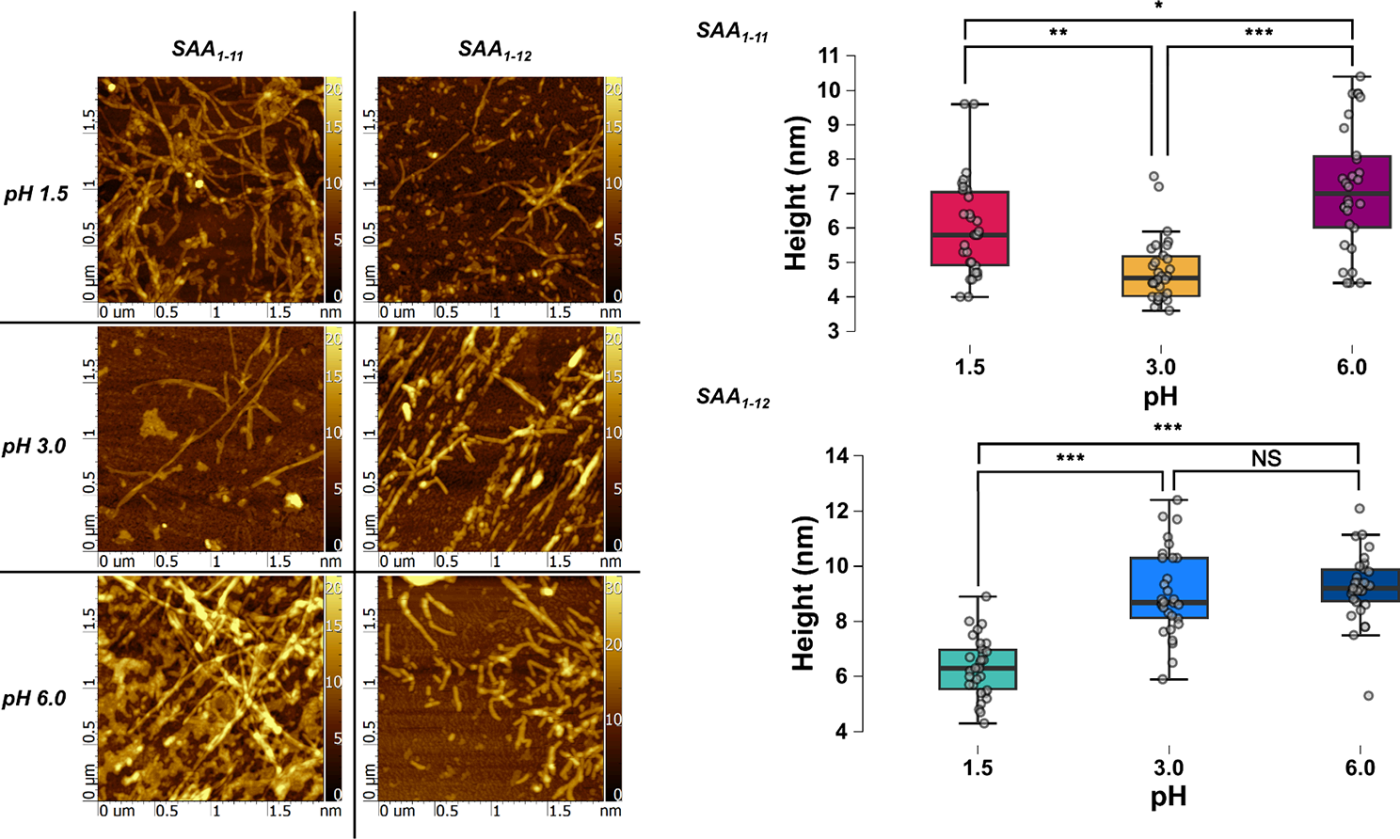
AFM images (left) and height profiles (right) of RSFFSFLGEAF (SAA_1–11_) and RSFFSFLGEAFD (SAA_1–12_) fibrils formed at pH 1.5, 3.0, and 6.0. Significance calculated utilizing one-way ANOVA with Tukey’s post hoc test. NS is a nonsignificant difference, and *p < 0.05, **p < 0.01, and ***p < 0.001.

RSFFSFLGEAF fibrils formed at the peptide isoelectric point (pH 6.0) also have a positive peak at 1616 cm^–1^ with a split of the negative peak to two centered around 1632 and 1650 cm^–1^, which points to some conformational changes of the RSFFSFLGEAF fibrils grown at this pH compared to the fibrils formed at pH 3.0. These conclusions are further supported by the observed difference in the height of these fibrils compared with the height of RSFFSFLGEAF fibrils grown at both pH 1.5 and pH 3.0 (Figure 2).

VCD revealed that RSFFSFLGEAFD fibrils formed at pH 1.5 exhibited a near-mirror-image VCD spectrum of RSFFSFLGEAF aggregates grown at the same pH. In the acquired spectrum, we observed negative (1620 cm^–1^) and positive (1649 cm^–1^) bands in the amide I band (Figure 3). These results indicated that the presence of aspartic acid at the peptide C-terminus reversed the supramolecular chirality of fibrils adopted by the peptide. Indeed, microscopic analysis of these aggregates performed by Rubin and co-workers revealed the presence of right-handed fibrils.^10^

**Figure 3:**
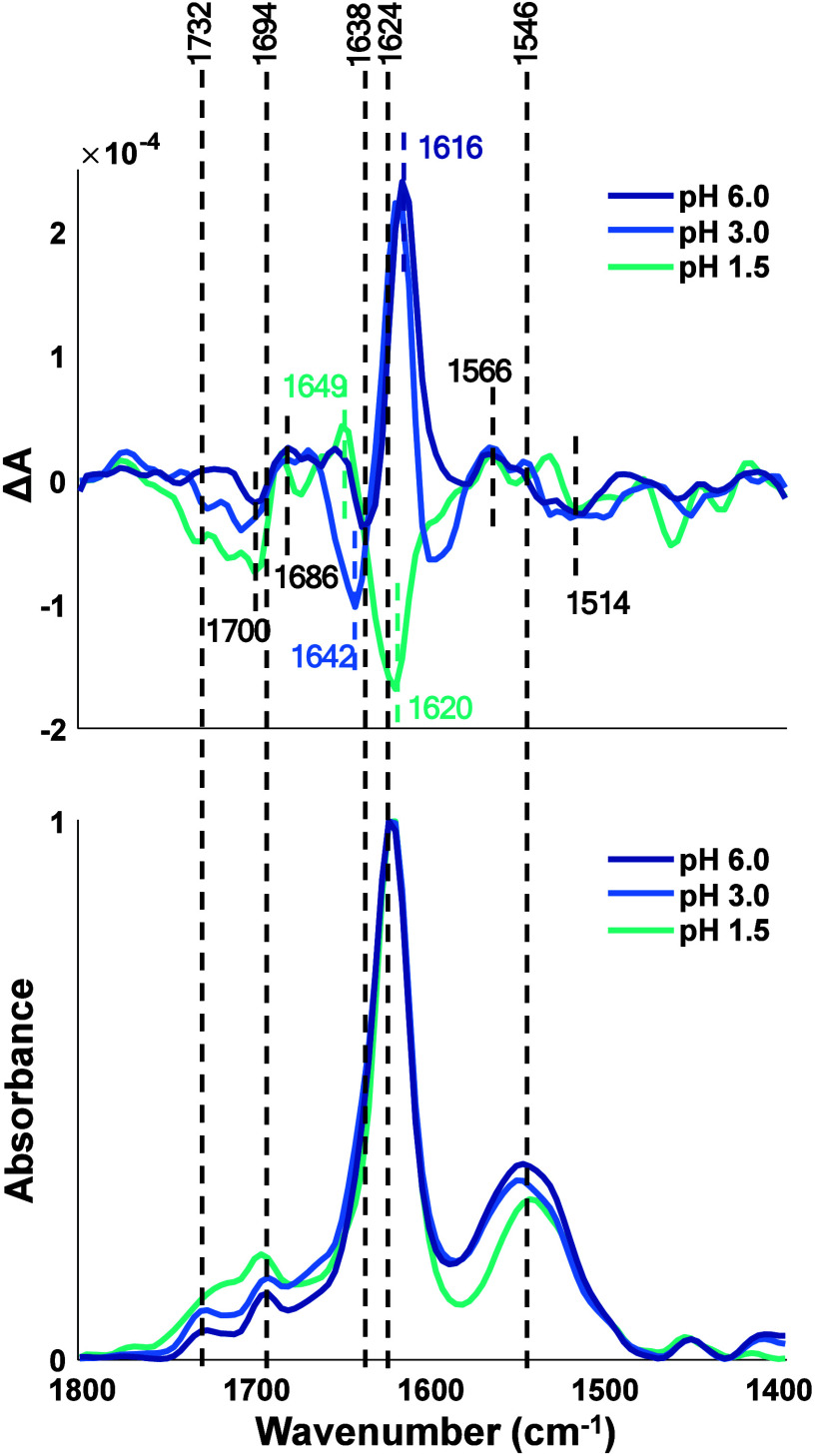
VCD (top) and IR (bottom) spectra acquired from RSFFSFLGEAFD fibrils formed at pH 1.5 (cyan), 3.0 (light blue), and 6.0 (dark blue).

Interestingly, RSFFSFLGEAFD fibrils formed at pH 3.0 and 6.0 had a reversed VCD compared with RSFFSFLGEAFD fibrils formed at pH 1.5. The VCD spectra acquired from these aggregates had an appositive peak at 1616 cm^–1^ and a negative peak at 1642 cm^–1^ (pH 3.0) and 1638 cm^–1^ (pH 6.0). We hypothesized that the flip of the VCD pattern could not be assigned solely to the deprotonation of the C-terminal carboxylic group because of the proximity of the side chain carboxylic group of aspartic acid. It is likely that the electrostatic interaction between the deprotonated C-terminal carboxylic acid and the aspartic acid side chain carboxylic acid results in the inversion of the supramolecular chirality of fibrils formed by such peptide at pH 3.0. It should be noted that RSFFSFLGEAFD fibrils formed at pH 3.0 were found to be significantly thicker compared to those aggregated at pH 1.5 (Figure 2). One can also expect that an additional negative charge on the C terminus of this peptide at pH 6.0 has a significant impact on the self-assembly of RSFFSFLGEAFD. Specifically, the electrostatic repulsion between the two negative charges likely alters peptide packing in the fibril structure, resulting in a shift of the VCD spectra. These conclusions are supported by the differences in the VCD spectra acquired from RSFFSFLGEAFD fibrils grown at pH 3.0 and 6.0 (Figure 3).

We utilized rat dopaminergic N27 cells to examine the cytotoxicity of both RSFFSFLGEAF and RSFFSFLGEAFD fibrils grown at pH 1.5. For this, both intact and sonicated fibrils were added to cells that reached 70% confluency. After 24 h of incubation, an ROS assay was used to determine the degree of cytotoxicity of both RSFFSFLGEAF and RSFFSFLGEAFD fibrils. We found that both protein aggregates exerted the same level of cytotoxicity (Figure 4). Importantly, the cytotoxicity levels of RSFFSFLGEAF and RSFFSFLGEAFD fibrils were substantially lower compared to those of insulin and lysozyme fibrils examined in our previous studies.^14,15^ It is also important to note that introduction of fibrils into cell culture media (pH 7.4) did not elicit a change in supramolecular chirality (Figure S1). On the basis of these results, we can conclude that supramolecular chirality of RSFFSFLGEAF and RSFFSFLGEAFD fibrils does not change their cytotoxic properties.

**Figure 4:**
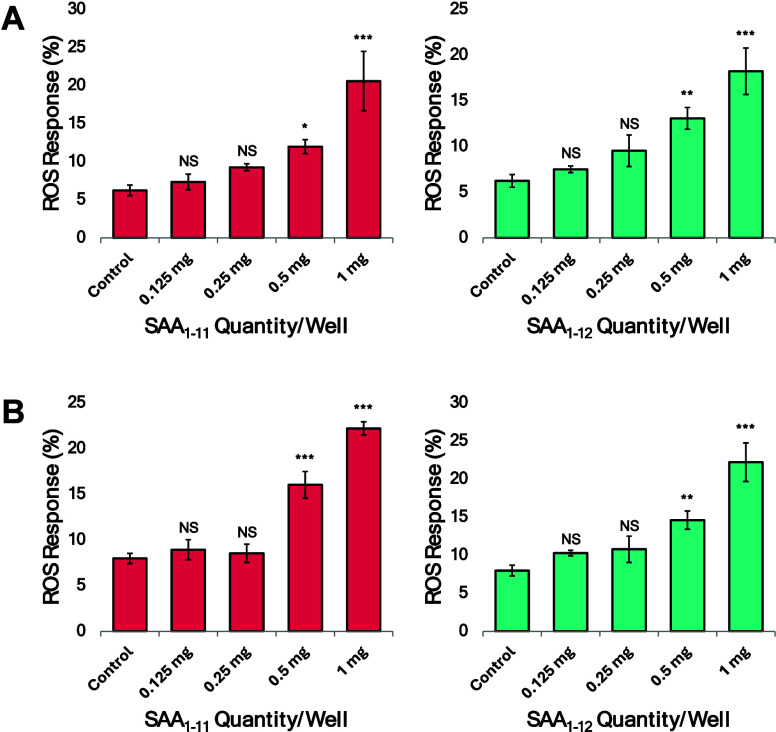
Cytotoxicity of intact (A) and sonicated (B) RSFFSFLGEAF (SAA_1–11_) and RSFFSFLGEAFD (SAA_1–12_) fibrils grown at pH 1.5. Bars represent the mean ± SD of *n* = 3 independent cultures. Significance calculated utilizing one-way ANOVA with Tukey’s post hoc test. NS is a nonsignificant difference, and *p ≤ 0.05, **p ≤ 0.01, and ***p ≤ 0.001.

Together, these presented findings demonstrate that the protonation state of a single amino acid in a peptide sequence can influence the supramolecular chiral assembly of the amyloid fibrils (Figure 5). Specifically, the coordination of negative charges induced by pH-dependent deprotonation in RSFFSFLGEAFD leads to a reversal of supramolecular chirality as pH is increased. This effect was not observed, however, in RSFFSFLGEAF fibrils, as they were formed in more acidic environments. Additionally, it was observed that RSFFSFLGEAFD fibrils exhibited a reversed supramolecular chirality as compared to RSFFSFLGEAF fibrils when both were formed at a pH below the p*K*_a_ of the C-terminus. The charges present within the peptide chain also strongly influence the backbone packing within the fibrils, as shown by the observed changes in fibril thickness and shifts in VCD spectra. These results point to the importance of primary structure as well as the proximity of ionizable groups within the peptide chain in the chiral self-assembly of amyloid fibrils. However, supramolecular chirality is not critical to fibril toxicity.

**Figure 5:**
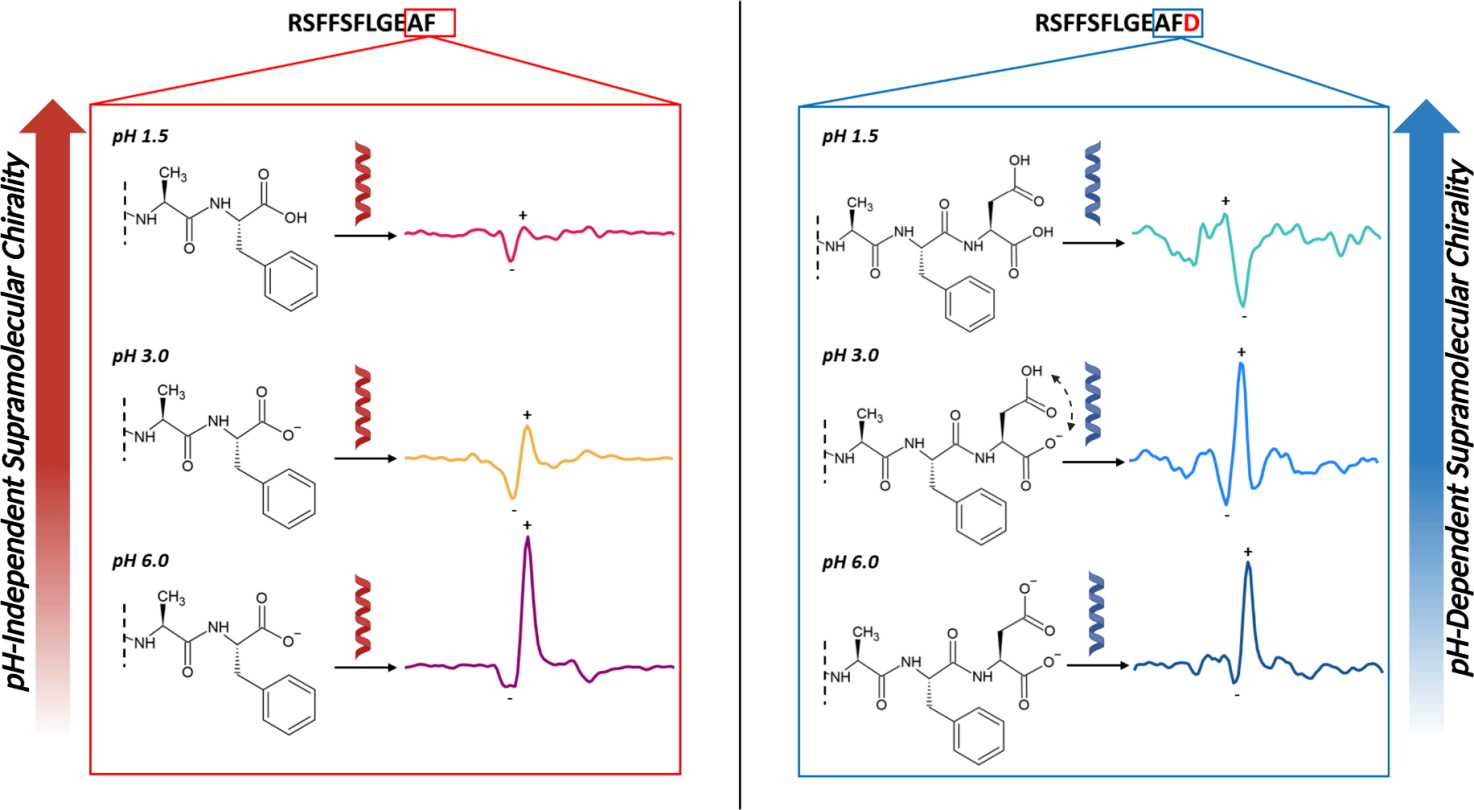
Schematic illustration of single amino acid-based control of fibril supramolecular chirality for RSFFSFLGEAF and RSFFSFLGEAFD peptides.

Limitations of the current study are as follows: The presented findings are only a proof-of-principle of such single-amino acid inversion of the fibril chirality. Therefore, generalization of our conclusions should be avoided until additional cases that support such peptide behavior are identified and studied. Additional studies are required to perform a careful and systematic analysis of such amino acid behavior in peptide-based self-assemblies.

## Supplementary Material



## Notes

Detailed description of sample preparation, spectra acquisition methods and instrumentation are shown; IR and VCD spectra of RSFFSFLGEAF and RSFFSFLGEAFD fibrils prepared at pH 1.5 and exchanged to pH 7.4 before measurement; IR and VCD spectra of RSFFSFLGEAF and RSFFSFLGEAFD prepared at pD 1.5, 3.0, or 6.0 in D_2_O; VCD spectra of RSFFSFLGEAF and RSFFSFLGEAFD fibrils prepared at pH 1.5, 3.0, or 6.0 and associated noise spectra; VCD spectra of RSFFSFLGEAF and RSFFSFLGEAFD fibrils with and without subtraction of water as well as the VCD spectrum of the water background (PDF)
