# Supplementary material for: Single Amino Acid-Based Control of Fibril Supramolecular Chirality
Source: J Phys Chem Lett. 2026 Jun 2;17(24):6637–41. doi: 10.1021/acs.jpclett.6c01104 (PMC13288619; doi:10.1021/acs.jpclett.6c01104)
Supplement: Supplementary file 1 [file jz6c01104_si_001.pdf]

Single Amino Acid-Based Control of Fibril Supramolecular Chirality

Jadon Sitton[1], and Dmitry Kurouski[*1,2]

[*]Email: dkurouski@tamu.edu

[1]Department of Biochemistry and Biophysics, Texas A&M University, College Station, Texas 77843, United States

[2]The Institute for Quantum Science and Engineering, Texas A&M University, College Station, Texas, 77843, United States

Supporting Information

Experimental Procedures:

Peptide sourcing: RSFFSFLGEAF and RSFFSFLGEAFD peptides were custom synthesized by AnaSpec Inc. (Fremont, CA, USA) with a free amine N-terminus and free acid C-terminus. Peptides were delivered as lyophilized powders and stored at -20°C.

Fibril preparation: RSFFSFLGEAF and RSFFSFLGEAFD peptides were fully dissolved in Milli-Q water at a concentration of 10 mg/ml before adjusting the pH of the solution to 1.5, 3.0, or 6.0 using HCl and NaOH. Peptide solutions were incubated for at least 24 hours at room temperature to allow fibrillation to occur. RSFFSFLGEAF and RSFFSFLGEAFD fibril samples were centrifuged at 13,000 RPM for 10 minutes at room temperature to harvest fibrils at the bottom of the sample tube.

Vibrational Circular Dichroism (VCD) and Infrared (IR) spectroscopy: VCD and IR spectra were acquired simultaneously using a BioTools Inc. (West palm Beach, FL, USA) ChiralIR-2X spectrometer equipped with an MCT detector and DualPEM at 8 cm^{-1} spectral resolution. For each measurement, ~20 µl of isolated fibrils were deposited in a BioTools Inc. BioCell with CaF_2 windows and a 6 µm pathlength. The BioCell was inserted into a BioTools Inc. SyncRoCell and rotated at a constant velocity about the IR beam axis to eliminate potential birefringence from the cell or sample. For each sample, IR and VCD spectra were acquired for 10 Blocks of 6,000 scans over the course of ~11 hours. Samples prepared in D_2O were measured under identical conditions to ensure water background did not convolute spectra (Figure S2&3). Noise spectra were simultaneously collected for each sample and compared to ensure VCD signal was above noise level (Figure S4). VCD and IR spectra from water and vapor were collected under identical conditions and subtracted from each respective sample spectra (Figure S5) using Thermo Fisher Scientific GRAMS/AI 7.0 software (Waltham, MA, USA). Each IR and VCD spectra were smoothed using the SavGol smoothing algorithm in EigenVector Research Inc. (Manson, WA, USA) PLS_Toolbox using a 7-point window and a polynomial order of 2. IR spectra were normalized to 1 and VCD spectra were normalized by the same normalization factor as IR. Figure construction and peak identification was performed in MathWorks (Natick, MA, USA) MATLAB and PLS_Toolbox. Measurements were repeated for each sample at least twice using independent samples to ensure reproducibility.

Atomic Force Microscopy (AFM) and fibril height measurement: Each sample was briefly diluted in Milli-Q water and immediately deposited onto a prewashed P-type silica substrate at a volume of 3.5 µl. Samples were incubated on the substrate for 15 minutes before aspirating any remaining solution by pipette and allowing the sample to ambiently dry at room temperature. Dry samples were then gently washed with Milli-Q water and dried under a gentle stream of nitrogen gas. Samples were then mounted onto magnetic sample discs using double-sided carbon tape. AFM images were recorded on an AIST-NT-HORIBA (Irvine, CA, USA) system in tapping mode and processed using the AIST-NT program. Height data was acquired by measuring the profile thickness of n=30 individual fibrils perpendicular to the fibril axis. Figure construction and statistical analysis were executed using JASP statistical software (Amsterdam, NLD).

Cell culture and toxicity assays: N27 rat dopaminergic neurons were purchased from Millipore (Burlington, MA, USA) and maintained in RPMI 1640 media supplemented with 10%

fetal bovine serum (FBS) and Normocin with incubation at 37°C under 5% CO_2. Early passage cells (<10 passages) were seeded in a cell culture treated 96-well plate in triplicates and incubated over night to achieve ~70% confluence. After the desired confluence was achieved, the cell media was replaced with fresh media and fibril samples were added at their respective quantities. Fibril samples were prepared identically to all other assays to allow for direct comparison between biophysical and cell-based assays. Intact fibrils were added directly to the cell media and sonicated fibril samples were sonicated in a water bath sonicator for 10 minutes at room temperature. Cells were incubated with fibril samples for 24 hours and then stained with CellROX Deep Red Dye from Invitrogen (Carlsbad, CA, USA) using the manufacturer's instructions. Toxicity was calculated using CellROX Deep Red fluorescence obtained from flow cytometry. Each assay was performed using n=3 independent cultures and repeated at least two times to ensure reproducibility.

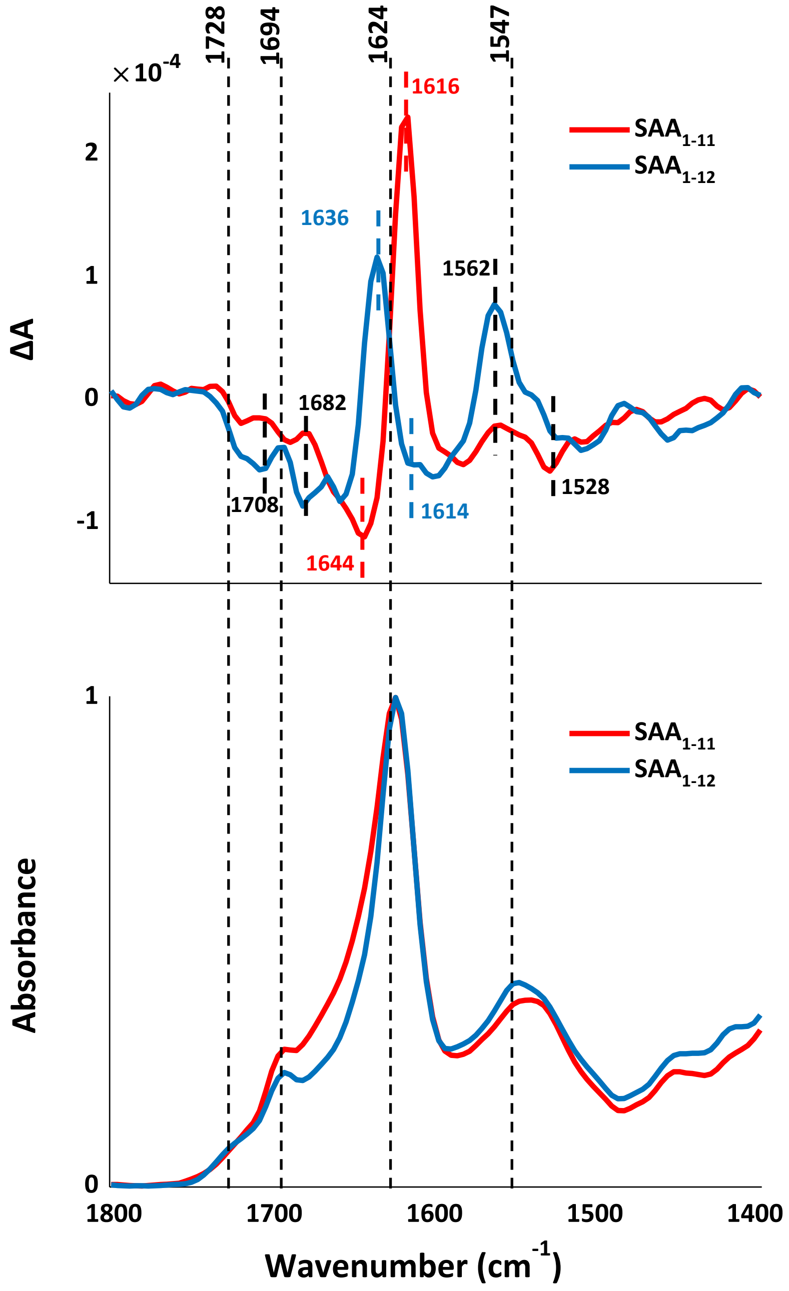

Figure S1: VCD (top) and IR (bottom) acquired from RSFFSFLGEAF (red) and RSFFSFLGEAFD (blue) fibrils formed at pH 1.5 and adjusted to pH 7.4 immediately before measurement.

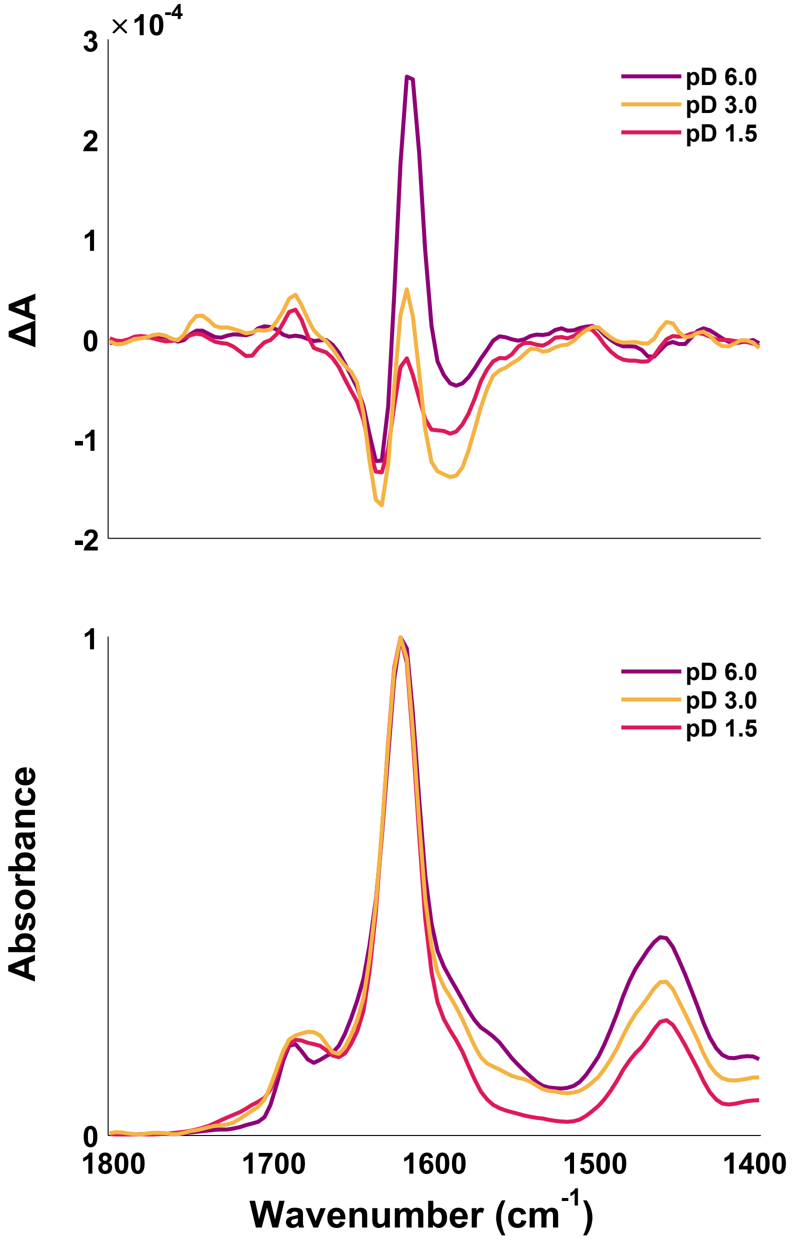

Figure S2: VCD (top) and IR (bottom) acquired from RSFFSFLGEAF fibrils formed in D_2O at pD 1.5 (pink), 3.0 (yellow), or 6.0 (purple).

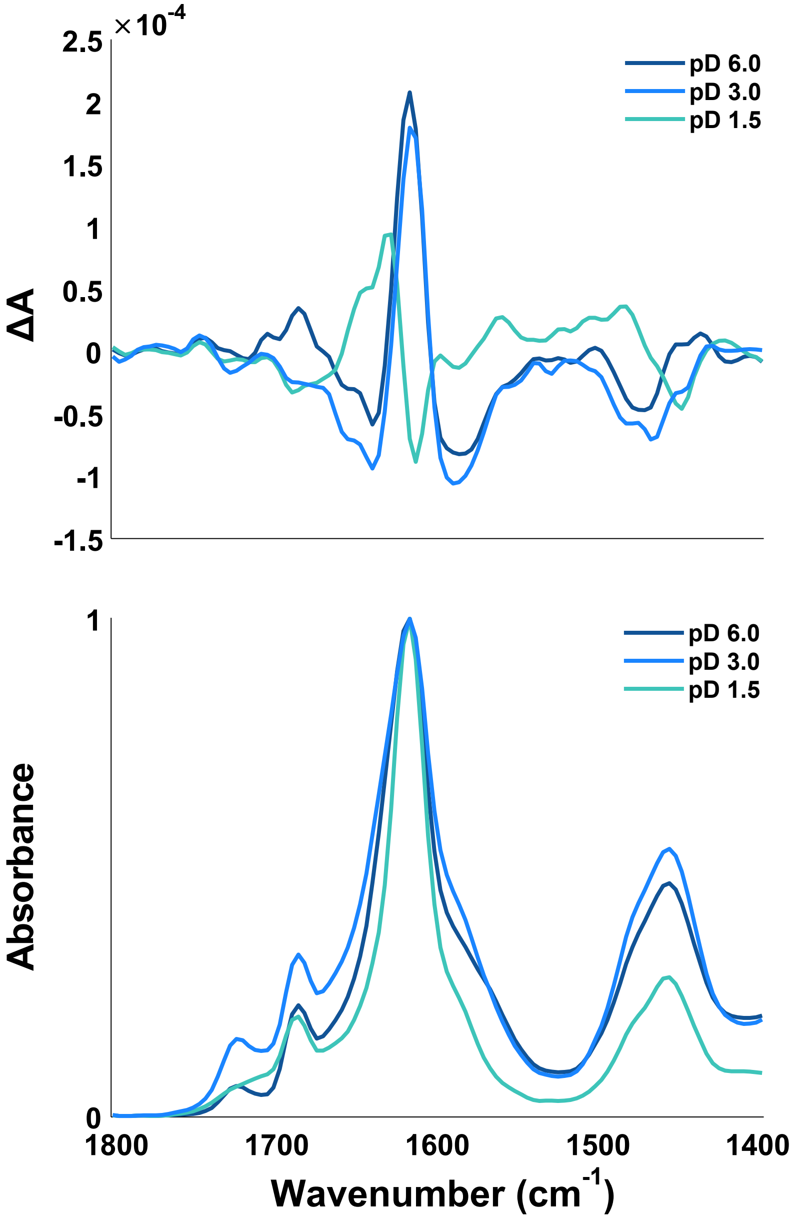

Figure S3: VCD (top) and IR (bottom) acquired from RSFFSFLGEAFD fibrils formed in D_2O at pD 1.5 (cyan), 3.0 (light blue), or 6.0 (dark).

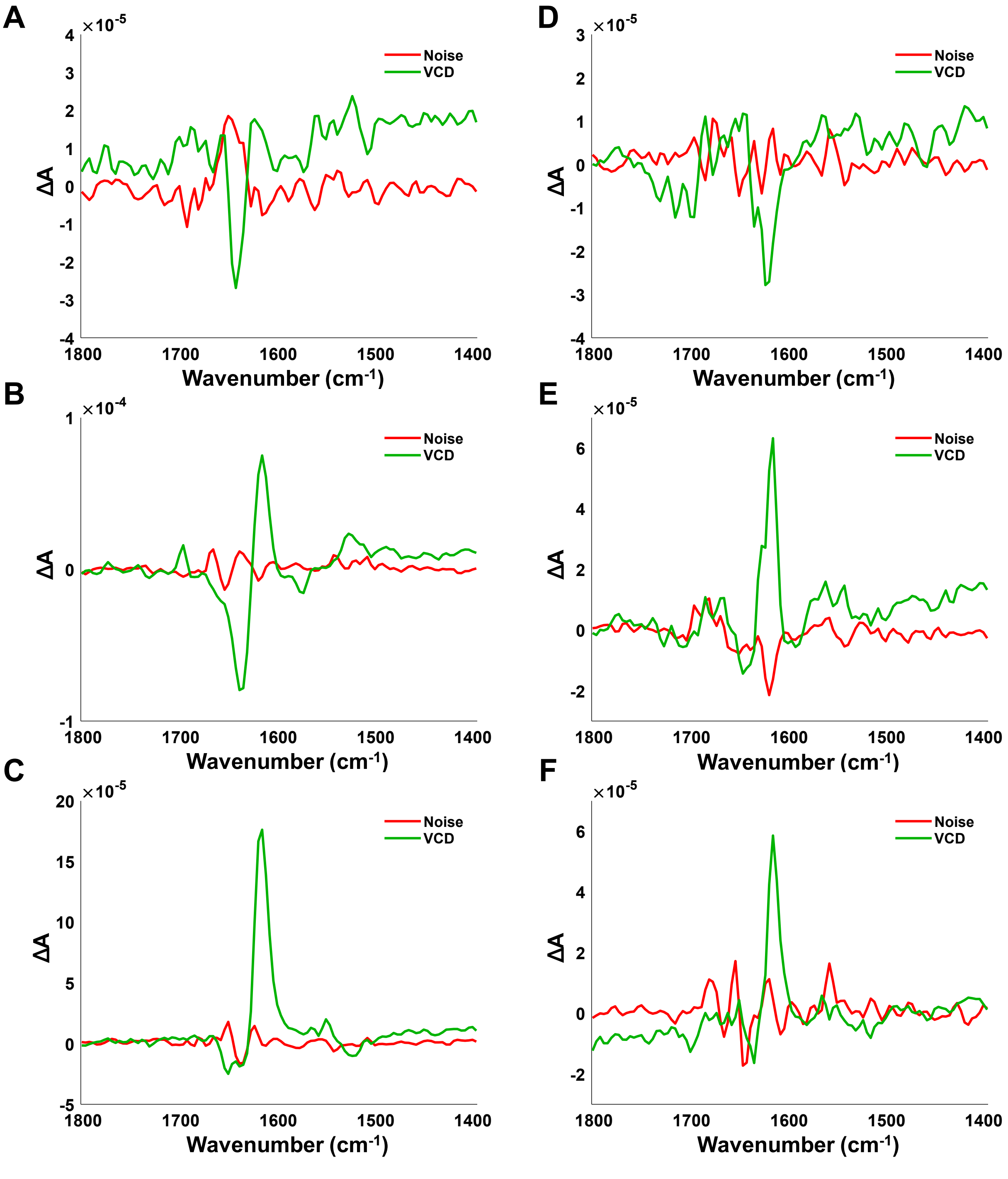

Figure S4: Noise (red) and VCD (green) spectra obtained from RSFFSFLGEAF (A-C) and RSFFSFLGEAFD (D-F) fibrils formed at pH 1.5 (A&D), 3.0 (B&E), or 6.0 (C&F).

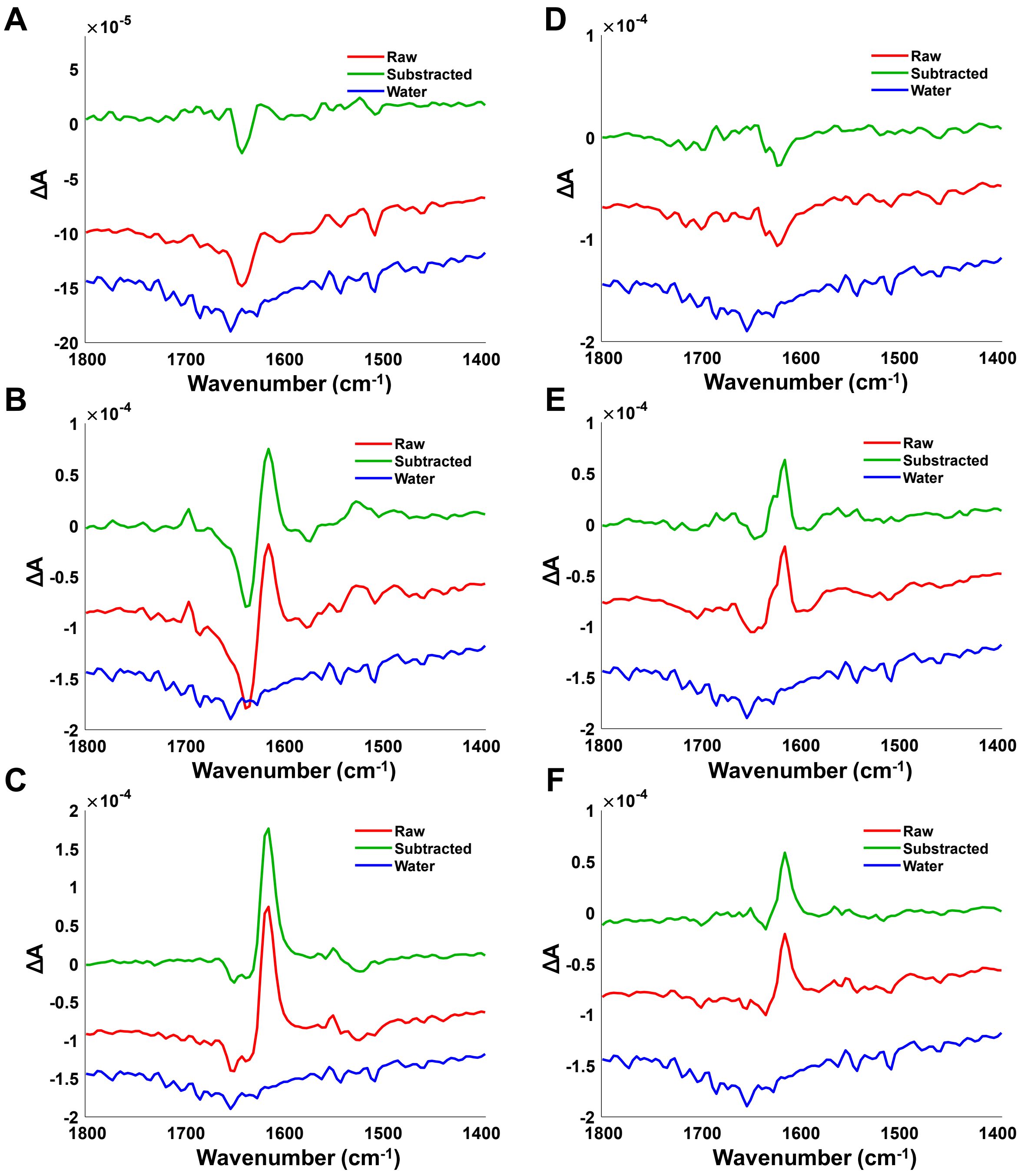

Figure S5: Raw (red) and subtracted (green) VCD spectra obtained from RSFFSFLGEAF (A-C) and RSFFSFLGEAFD (D-F) fibrils formed at pH 1.5 (A&D), 3.0 (B&E), or 6.0 (C&F), as well as spectra of water alone (blue).
